# Metabolomics biomarkers and the risk of overall mortality and ESRD in CKD: Results from the Progredir Cohort

**DOI:** 10.1371/journal.pone.0213764

**Published:** 2019-03-18

**Authors:** Silvia M. Titan, Gabriela Venturini, Kallyandra Padilha, Alessandra C. Goulart, Paulo A. Lotufo, Isabela J. Bensenor, Jose E. Krieger, Ravi I. Thadhani, Eugene P. Rhee, Alexandre C. Pereira

**Affiliations:** 1 Nephrology Division, University of Sao Paulo Medical School, Sao Paulo, SP, Brazil; 2 Laboratory of Genetics and Molecular Cardiology, Heart Institute, University of Sao Paulo Medical School, Sao Paulo, SP, Brazil; 3 Epidemiological and Clinical Research Center, University Hospital, Sao Paulo University, Sao Paulo, Brazil; 4 Division of Nephrology, Department of Medicine, Massachusetts General Hospital, Boston, MA, United States of America; 5 Vice Dean of Research, Cedars-Sinai Medical Center, Los Angeles, CA, United States of America; 6 Division of Endocrinology, Department of Medicine, Massachusetts General Hospital, Boston, MA, United States of America; Postgraduate Medical Institute, INDIA

## Abstract

**Introduction:**

Studies on metabolomics and CKD have primarily addressed CKD incidence defined as a decline on eGFR or appearance of albuminuria in the general population, with very few evaluating hard outcomes. In the present study, we investigated the association between metabolites and mortality and ESRD in a CKD cohort.

**Setting and methods:**

Data on 454 participants of the Progredir Cohort Study, Sao Paulo, Brazil were used. Metabolomics was performed by GC-MS (Agilent MassHunter) and metabolites were identified using Agilent Fiehn GC/MS and NIST libraries. After excluding metabolites present in <50% of participants, 293 metabolites were analyzed. An FDR q value <0.05 criteria was applied in Cox models on the composite outcome (mortality or incident renal replacement therapy) adjusted for batch effect, resulting in 34 metabolites associated with the outcome. Multivariable-adjusted Cox models were then built for the composite outcome, death, and ESRD incident events. Competing risk analysis was also performed for ESRD.

**Results:**

Mean age was 68±12y, mean eGFR-CKDEPI was 38.4±14.6 ml/min/1.73m^2^ and 57% were diabetic. After adjustments (GC-MS batch, sex, age, DM and eGFR), 18 metabolites remained significantly associated with the composite outcome. Nine metabolites were independently associated with death: D-malic acid (HR 1.84, 95%CI 1.32–2.56, p = 0.0003), acetohydroxamic acid (HR 1.90, 95%CI 1.30–2.78, p = 0.0008), butanoic acid (HR 1.59, 95%CI 1.17–2.15, p = 0.003), and docosahexaenoic acid (HR 0.58, 95%CI 0.39–0.88, p = 0.009), among the top associations. Lactose (SHR 1.49, 95%CI 1.04–2.12, p = 0.03), 2-O-glycerol-α-D-galactopyranoside (SHR 1.76, 95%CI 1.06–2.92, p = 0.03), and tyrosine (SHR 0.52, 95%CI 0.31–0.88, p = 0.02) were associated to ESRD risk, while D-threitol, mannitol and myo-inositol presented strong borderline associations.

**Conclusion:**

Our results identify specific metabolites related to hard outcomes in a CKD population. These findings point to the need of further exploration of these metabolites as biomarkers in CKD and the understanding of the underlying biological mechanisms related to the observed associations.

## Introduction

Metabolomics has emerged as a new technology for the study of biomarkers and pathways involved in diseases. In the setting of CKD, some recent epidemiological studies have evaluated the role of metabolomics-derived biomarkers. Using a cross-sectional design, Sekula *et al* initially reported metabolites related to eGFR in the KORA F4 Study (n = 1735) and replicated in the Twins-UK Study (n = 1164), with pseudouridine, C-mannosyltryptophan, N-acetylalanine, erythronate, myo-inositol and N-acetylcarnosine emerging as the top associations [1]. In the same publication, the authors showed that C-mannosyltryptophan, pseudouridine and O-sulfo-L-tyrosyne were also significantly associated with the risk of incident CKD in the KORA Study. Similar findings were reported by Solini *et al* among 285 type 2 diabetes patients in relation to incident CKD [2]. KORA F4 and Twins Study populations were also used in a cross-sectional study using targeted metabolomics [3], with results showing glutarylcarnitine as the top association with eGFR. Yu *et al* showed that 5-oxoproline and 1,5-anhydroglucitol were independent predictors of incident CKD in an African American population [4], and the association to 1,5-anhydroglucitol was replicated by our group in a diabetic kidney disease population [5]. Rhee et al showed that 9 metabolites were related to the risk of incident CKD in the Framingham Heart Study [6]. Using information from 5 of them (kynurenic acid, xanthosine, 5-hydroxyindoleacetic acid, kynurenine and citrulline) improved the prediction accuracy for incident CKD events compared to a model based only on traditional risk factors. Arginine, methionine and threonine were shown to be potential indicators of renal metabolic function and biomarkers of renal prognosis in a case-control study comparing participants in a CKD cohort presenting a fast decline on eGFR in comparison to those showing no decline or a low decline [7].

However, few studies have evaluated metabolomics in relation to incident ESRD or other hard outcome. In a case-control study, Niewczas et al showed biomarkers related to ESRD in type 2 diabetes [8]. The same group reported 7 metabolites (C-glycosyltryptophan, pseudouridine, O-sulfotyrosine, N-acetylthreonine, N-acetylserine, N6-carbamoylthreonyladenosine and N6-acetyllysine) associated to ESRD in type 1 diabetes with CKD at baseline [9].

While suggesting interesting insights such as potential novel biomarkers of kidney function, the role of kidney in the metabolism of aminoacids, the role of some metabolites in neurological and nutritional abnormalities seen in uremia, and new biomarkers for predicting hard outcomes, metabolomics studies in CKD are still few and with diverse results. In addition, most studies were performed in populations free of CKD at baseline. Although this may be an important strategy for deriving biomarkers for early disease, this strategy may fall short to identify metabolites associated to clinical events when CKD is already established.

In this study, we investigated the relationship between metabolomic biomarkers measured by GC-MS (Gas Chromatography combined with Mass Spectrometry) and overall mortality and incident ESRD in the Progredir Cohort Study, an ongoing CKD cohort in Sao Paulo, Brazil.

## Methods

### Sample study and outcomes

Details on the Progredir Cohort Study recruitment and baseline collection have been published elsewhere [10]. Briefly, patients from the Hospital das Clínicas Outpatient Clinic, a quaternary hospital in Sao Paulo, Brazil, were invited to participate in the study. Outpatient records were reviewed and all patients with age ≥ 30 years-old and at least two measurements of creatinine (with a minimum interval of 3 months) ≥1.6 mg/dL for men and ≥1.4 mg/dL for women were considered potential candidates. Patients attending oncology, psychiatry, urology, HIV/AIDS, viral hepatitis and glomerulonephritis services were excluded. The remaining candidates were then contacted by phone and invited to participate if no exclusion criteria were met (hospitalization or acute myocardial infarction in the last 6 months, autoimmune diseases, pregnancy, psychiatric diseases, ongoing chemo or immunosuppressive therapy, ongoing renal replacement therapy, glomerulonephritis, HIV/AIDS infection, hepatitis C and B, and any previous organ transplantation). Recruitment took place from March 2012 to December 2013, and 454 participants were enrolled. The study was approved by two local Ethics Committees (Ethics in Research Committee–Universitary Hospital, Sao Paulo University, n° 11147/11; and Ethics Commission for Analysis of Research Projects, Hospital das Clínicas, Medical School, Sao Paulo University, n° 0798/11) and written informed consent was obtained from all participants.

Participants were scheduled for a one-day visit in the research center for interviews and clinical exams according to standard protocols performed by trained personal under strict quality control. Overnight fasting blood samples, 24hour and spot urine were collected. Urine and blood aliquots were readily prepared and stored in liquid nitrogen. Diabetes was defined as previous medical history of diabetes, use of medication to treat diabetes, fasting plasma glucose ≥126 mg/dl, HbA1C ≥6.5% or a 2-hour plasma glucose ≥200 mg/dl after a 75g-glucose tolerance test. Glomerular filtration rate was estimated by the CKD-EPI equation [11] and albumin-to-creatinine ratio (ACR) was performed in a morning spot urine. Laboratory data were determined using conventional techniques [10].

Follow-up is ongoing and made through annual telephone interviews including questions on death, hospitalizations, and need of renal replacement therapy (RRT). Vital status is investigated periodically by a hot-pursuit strategy [12]. Mortality information is confirmed by official death certificates with the collaboration of several health offices (PRO-AIM, Fundação SEADE and Brazilian National Mortality Registry). RRT is ascertained through the city and state´s Registries (Sao Paulo State Registry of Dialysis and Kidney Transplantation, Sao Paulo City Registry of Dialysis and Kidney Transplantation).

### Metabolomics

Metabolomics was performed according to a standard protocol with few modifications [13]. Serum samples were thawed on ice at 4°C for 30–60 min. Metabolites from each aliquot of plasma (70μL) were extracted with a 300μL solvent mixture of acetonitrile, isopropanol and deionized water (3:3:2 v/v) and spiked with a 5μL internal standard solution (RTL). After vortexing for 15 sec, the mixture was centrifuged for 15 min at 15.800xg at 4°C. Supernatant (320μL) was transferred to a new microcentrifuge tube, followed by lyophilization in a Speedvac concentrator for 18 hours. Subsequently, the residue was suspended in 50μL methoxyamine in pyridine (Sigma-Aldrich) solution (40mg/mL), 3μL of FAME (Fatty acid methyl ester–Sigma-Aldrich) was added, and the mixture was vortexed for 3 min. This methoximation reaction was performed at room temperature for 16h, followed by trimethylsilylation for 1h adding 100μL MSTFA (N-methyl-N-trimethylsilyltrifluoroacetamine) with 1% TMCS (trimethylchlorosilane) (Sigma-Aldrich). After derivatization, 1μL of this derivative was used for GC-MS in Agilent 7890B GC system operated in splitless mode. A DB5-MS + 10m Duraguard capillary column (Agilent 122-5532G) within which helium carrier gas flowed at a rate of 1.1mL min^-1^, was applied for metabolite separation. The injector temperature was set at 250°C. The column temperature was held at 60°C for 1 min, and then increased to 310°C at a rate of 10°C/min during 37 minutes. The column effluent was introduced into the ion source of an Agilent 5977A mass selective detector. The detector operated in the electron impact ionization mode (70 eV) and mass spectra were recorded after a solvent delay of 6.5 min with 3 scans per second. The MS quadrupole temperature was set at 180°C and the ion source temperature was set at 280°C. Each sample was analyzed in three technical replicates. We used blank (serum replaced by deionized water) and quality control (serum pool) samples every day to verify the existence of impurities in the reagents and equipment contamination, as well as to check the sensitivity and compliance of the injector system, respectively.

Identification of compounds was made comparing the mass spectra and retention time (RT) of all detected compounds with the Agilent Fiehn GC/MS Metabolomics RTL Library (version A.02.02) and the National Institute of Standards and Technology (NIST) library 11 (2014) using Unknowns—Agilent MassHunter Workstation Quantitative Analysis (version B.06.00). Retention time and electron ionization spectra were used for metabolite identification. Absolute retention times were locked to the internal standard d27-myristic acid 3mg/mL (Product # 366889; Sigma- Aldrich; RT of the locking standard is 16.752 minutes) using the RTL system provided in Agilent’s MassHunter software.

### Statistical analysis

Metabolomics data were available for 450 of the 454 participants. In addition, 1 participant was lost to follow-up and renal replacement therapy could not be ascertained for 5 participants. Deaths and ESRD were ascertained up to May-2017. Censoring date was defined as the last day of contact.

Initially, 10940 metabolites were identified from the spectrograms in at least one sample. Only those identified in at least 50% of the samples were kept for analysis (n = 293 metabolites, later reduced to 265 after exclusion of column contaminants and internal standards). For survival analysis, we first performed Cox regression models on a composite outcome of overall death and incident renal replacement therapy (n = 129) adjusted only for GC-MS batch to select metabolites with a false discovery rate (FDR) q value > 0.05. This procedure selected 34 metabolites. We then adjusted multivariable models for each selected metabolite on the risk of the composite outcome. These models were adjusted for the effect of traditional risk factors (age, sex, diabetes, SBP, and eGFR at baseline). We also evaluated hazard risks of events of all-cause mortality alone (n = 93) and incident ESRD alone (n = 36). Since mortality imposes an important competing risk on the risk of ESRD in a CKD population, we repeated the survival analyses for ESRD using unadjusted and adjusted Fine and Gray models. We assessed the performance on ROC curves of traditional models (based on age, sex, DM, SBP, baseline eGFR and smoking) versus models with added selected metabolites.

Finally, pathway was explored by the construction of correlation matrix and pathway analysis. In addition, by using the 34 selected metabolites we have derived metabolite networks based on partial correlations using a modification of a graphical Gaussian model, stratified by biochemical class.

Statistical analyses were done using SPSS (version 20.0) and R (“survival” and “cmprsk” packages). For pathway analysis we have used MetaboloaAnalyst (http://www.metaboanalyst.ca/) using KEGG *homo sapiens* library and hypergeometric test adjusted for multiple comparisons (FDR<0.05). For the metabolite network, we used a model implemented in the “mgm” R package (https://cran.r-project.org/web/packages/mgm/index.html).

## Results

In Table 1, we show the baseline characteristics of the 454 participants of the Progredir Cohort Study. The participants had a mean age of 67.5 years and presented a mean eGFR of 38.4 ±14.6 ml/min/1.73m^2^, at study entry. The majority of study subjects were male (63.2%), had hypertension (90.1%) and/or diabetes (56.8%), and 30% reported a previous myocardial infarction and 16% a previous stroke. Over a mean follow-up time of 3 years, 129 events of death and renal replacement therapy occurred and differences in baseline variables according to these events are also shown in Table 1. As expected, age, baseline renal function, diabetes, previous myocardial infarction and systolic blood pressure (SBP) were different among groups.

**Table 1:** Baseline characteristics of all participants and according to the composite outcome in the Progredir Cohort.

	All n = 454	No events n = 319	Death or RRT n = 129	p*
Age (years; mean / std)	67.5 (11.9)	66.8 (11.8)	69.3 (11.6)	0.05
Sex (men; n / %)	287 (63.2%)	200 (62.7%)	84 (65.1%)	0.63
Race (white; n/ %)	300 (66.1%)	219 (69.5%)	80 (62.5%)	0.15
Hypertension (n/ %)	409 (90.1%)	282 (89.2%)	122 (94.6%)	0.08
Diabetes (n/%)	257 (56.6%)	172 (53.9%)	82 (63.6%)	0.06
Previous myocardial infarction (n/ %)	147 (32.4%)	95 (30.3%)	51 (39.5%)	0.06
Previous stroke (n/ %)	73 (16.1%)	48 (15.4%)	23 (18.5%)	0.43
Smoking (current or previous; n / %)	269 (59.3%)	184 (57.7%)	83 (64.3%)	0.19
SBP (mmHg; mean / std)	140 (24)	139 (22)	144 (28)	0.05
DBP (mmHg; mean / std)	76 (13)	76 (12)	76 (15)	0.90
Body-mass index (mean / std)	29.4 (5.4)	29.3 (5.0)	29.4 (6.4)	0.89
Waist-to-hip ratio (mean / std)	0.97 (0.10)	0.97 (0.11)	0.98 (0.07)	0.89
Potassium (mEq/L; mean / std)	4.6 (0.5)	4.6 (0.5)	4.7 (0.6)	0.02
Urea (mg/dL; median/ IQR)	69 (54–89)	65 (53–84)	81 (64–107)	<0.001
Creatinine (mg/dL;median / IQR)	1.7 (1.4–2.1)	1.6 (1.4–1.9)	2.1 (1.5–2.8)	<0.001
Albuminuria (mg/g creatinine; median / IQR)	80 (15–640)	54 (11–366)	344 (47–1529)	<0.001
eGFR-CKDEPI (mL/min/1.73 m^2^; mean / std)	38.4 (14.6)	41 (14)	32 (15)	<0.0001
Phosphorus (mg/dL; mean / std)	3.6 (0.6)	3.6 (0.6)	3.9 (0.7)	<0.0001
Calcium (mg/dL; mean / std)	9.6 (0.6)	9.6 (0.5)	9.5 (0.6)	0.004
Parathormone (pg/mL; median / IQR)	93 (64–143)	85 (57–126)	126 (84–224)	<0.001
Glycemia (mg/dL; median / IQR)	104 (95–126)	103 (95–125)	106 (94–132)	0.30
Glycated hemoglobin (%; median / IQR)	6.2 (5.8–7.2)	6.1 (5.7–7.0)	6.6 (5.9–7.6)	0.006
Total cholesterol (mg/dL mean / std)	169 (40)	170 (40)	165 (41)	0.28
LDL-cholesterol (mg/dL; mean / std)	91 (32)	92 (33)	89 (31)	0.39
HDL-cholesterol (mg/dL; mean / std)	46 (14)	46 (15)	45 (12)	0.57
Triglycerides (mg/dL; median / IQR)	142 (99–192)	142 (102–192)	137 (92–189)	0.41
Bicarbonate (mmol/L; mean / std)	25.6 (2.9)	25.6 (2.9)	25.6 (3.1)	0.91
Hemoglobin (g/dL; mean / std)	13.1 (1.9)	13.4 (1.9)	12.5 (1.8)	<0.0001
Albumin (mg/dL; mean / std)	4.3 (0.3)	4.3 (0.3)	4.2 (0.4)	<0.0001

* t test for gaussian, Mann-Whitney for non-gaussian and chi square for categorical variables.

Initially, the relationship between metabolites and baseline eGFR was evaluated and these results were published elsewhere [14]. While some metabolites presented high inverse associations to CKD-EPI eGFR, other metabolites, such as tyrosine and glycerol, showed decreasing levels as eGFR goes down [14].

For the survival analysis proposed here, we initially selected metabolites that reached an FDR q value <0.05 in Cox proportional hazard models on the composite outcome adjusted only for GC-MS batch effect, an approach that left 34 metabolites for further analysis, shown in Table 2 (a full list of the 265 metabolites and estimates is provided as S1 Table). Those 34 selected metabolites were also evaluated in *homo sapiens* pathway analysis (S2 Table). Although not reaching significant values for multiple comparisons, aminoacyl-tRNA biosynthesis, galactose metabolism, pentose phosphate pathway and tyrosine metabolism appeared as enriched considering raw p values.

**Table 2:** List of metabolites significantly (FDR<0.05) related to composite outcome (n = 129) in Cox regression models adjusted only for batch.

Metabolite	Biochemical class	HR*	p value	FDR q values
Lactose	Carbohydrates and carbohydrate conjugates	1.57	8.30E-12	2.43E-09
D-threitol	Carbohydrates and carbohydrate conjugates	2.46	9.09E-11	1.33E-08
Pseudouridine	Nucleoside and nucleotide analogues (class)	2.05	3.15E-09	2.58E-07
Butanoic acid	Fatty acids and conjugates	1.83	3.52E-09	2.58E-07
D-mannitol	Carbohydrates and carbohydrate conjugates	1.36	9.17E-08	5.37E-06
Trans-aconitic acid	Tricarboxylic acids and derivatives	2.07	4.41E-07	2.15E-05
Acetohydroxamic acid	Carboxylic acid derivatives	2.06	1.56E-06	6.53E-05
Galactonic acid	Medium-chain hydroxy acids and derivatives	1.62	5.11E-06	1.87E-04
Myo-inositol	Alcohols and polyols	1.97	6.09E-06	1.98E-04
L-threonine	Amino acids, peptides, and analogues	0.60	7.78E-06	2.28E-04
2-O-Glycerol-α-D-galactopyranoside	Carbohydrates and carbohydrate conjugates	1.54	3.78E-05	1.01E-03
Galacturonic acid	Carbohydrates and carbohydrate conjugates	1.57	5.34E-05	1.30E-03
L-glutamine	Amino acids, peptides, and analogues	1.65	6.58E-05	1.39E-03
Xylitol	Carbohydrates and carbohydrate conjugates	1.51	6.64E-05	1.39E-03
Gluconic acid	Carbohydrates and carbohydrate conjugates	1.79	1.29E-04	2.52E-03
5-hydroxyindol	Hydroxyindoles	1.37	1.59E-04	2.90E-03
Unidentified m/z 405	-	1.56	1.71E-04	2.95E-03
Ribose	Carbohydrates and carbohydrate conjugates	1.40	2.33E-04	3.80E-03
p-Cresol glucuronide	Arylsulfates	1.30	2.74E-04	4.22E-03
Tyrosine	Amino acids, peptides, and analogues	0.67	3.26E-04	4.77E-03
(S)-3,4-Dihydroxybutyric acid	Beta hydroxy acids and derivatives	1.74	3.83E-04	5.34E-03
L-serine	Amino acids, peptides, and analogues	1.56	5.82E-04	7.74E-03
p-Hydroxyphenylacetic acid	1-hydroxy-2-unsubstituted benzenoids	1.28	9.19E-04	1.17E-02
Phenol	1-hydroxy-4-unsubstituted benzenoids	1.27	1.14E-03	1.40E-02
Eicosapentaenoic acid	Fatty acids and conjugates	1.39	1.25E-03	1.46E-02
Unidentified m/z 273	-	1.93	1.96E-03	2.21E-02
Ribonic acid	Carbohydrates and carbohydrate conjugates	1.51	2.21E-03	2.40E-02
D-malic acid	Fatty acids and conjugates	1.52	2.54E-03	2.66E-02
Unidentified m/z 296	-	1.57	3.15E-03	3.18E-02
L-proline	Amino acids, peptides, and analogues	0.73	4.33E-03	4.23E-02
Acetamide	Carboximidic acids	1.56	5.16E-03	4.87E-02
p-cresol	Cresols	1.34	5.32E-03	4.87E-02
Doconexent (docosahexaenoic acid)	Fatty acids and conjugates	0.63	5.64E-03	5.01E-02
Threonic acid	Carbohydrates and carbohydrate conjugates	1.52	6.44E-03	5.55E-02

Models adjusted only for batch.

* HR per 1 unit log base 2.

Next, Cox regression models for these 34 metabolites on the composite outcome were rebuilt, now adjusting for the effect of sex, age, baseline eGFR, diabetes, and batch. The variables significantly associated with the composite outcome after these adjustments are shown in Table 3 and the correlation between these metabolites is provided in S3 Table. These metabolites, tested either as combination of top associations or combination of metabolites selected by stepwise models, did not improve the performance of a model based on traditional cardiovascular risk factors (age, sex, diabetes, SBP, baseline eGFR and smoking) for the prediction of mortality and/or ESRD events (data not shown).

**Table 3:** Adjusted Cox regression models on the risk of the composite outcome (n = 129) in the Progredir Cohort Study.

Composite outcome—Cox adj. batch, sex, age, eGFR and DM				
	HR*	95%CI HR		p
Lactose	1.37	1.18	1.60	.0001
Acetohydroxamic acid	1.86	1.34	2.57	.0002
D-threitol	1.80	1.25	2.59	.002
Doconexent (docosahexaenoic acid)	0.57	0.41	0.81	.002
Butanoic acid	1.48	1.14	1.92	.003
D-mannitol	1.21	1.07	1.37	.003
Trans-aconitic acid	1.65	1.17	2.32	.004
Pseudo uridine	1.60	1.14	2.25	.006
L-glutamine	1.42	1.08	1.87	.01
L-threonine	0.76	0.61	0.95	.01
Eicosapentaenoic acid	1.29	1.05	1.60	.02
Ribose	1.25	1.03	1.52	.02
D-malic acid	1.36	1.03	1.79	.03
Unindentified m/z 273	1.40	1.03	1.90	.03
L-serine	1.33	1.02	1.72	.03
p-Cresol glucuronide	1.17	1.01	1.35	.042
galacturonic acid	1.29	1.00	1.65	.048
2-O-Glycerol-α-d-galactopyranoside	1.27	1.00	1.62	.049

Models were adjusted for batch, sex, age, eGFR and diabetes.

* HR per 1 unit log base 2

In order to better understand the relationship between associated markers with the incidence of combined endpoints we have constructed a Gaussian graphical model containing all 34 pre-selected metabolites and their identified isoforms, age, sex, diabetes, calculated glomerular filtration rate and the presence of the combined outcome. In S1 Fig. we depict the relationship between the partial correlations of all these variables together.

We then repeated these models separating the events of mortality only (Table 4) and ESRD only (Table 5). As can be seen in Table 4, D-malic acid, acetohydroxamic acid, butanoic acid, ribose, glutamine, trans-aconitic acid, lactose and an unidentified molecule (m/z 273) were all positively related to the risk of death, while docosahexaenoic acid was inversely associated to overall mortality.

**Table 4:** Adjusted Cox regression models on the risk of overall death (n = 93) in the Progredir Cohort Study.

Death—Cox adj batch, sex, age, eGFR and DM				
	HR*	95%CI HR		p
D-malic acid	1.84	1.32	2.56	.0003
Acetohydroxamic acid	1.90	1.30	2.78	.0008
Butanoic acid	1.59	1.17	2.15	.003
Doconexent (docosahexaenoic acid)	0.58	0.39	0.88	.009
Ribose	1.26	1.01	1.57	.04
L-glutamine	1.40	1.01	1.94	.04
Trans-aconitic acid	1.54	1.01	2.36	.04
Lactose	1.21	1.00	1.46	.05
Unindentified m/z 273	1.45	1.00	2.11	.05

Models were adjusted for batch, sex,age, eGFR and DM.

* HR per 1 unit log base 2

**Table 5:** Adjusted Cox regression and Fine-Gray models (subdistribution analysis of competing risks) on the risk of ESRD (n = 36) in the Progredir Cohort Study.

	Cox			Fine and Gray		
Metabolite	HR*	95%CI HR	p	SHR*	95%CI SHR	P value
Lactose	1.68	1.21–2.34	.002	1.49	1.04–2.12	0.03
2-O-Glycerol-α-D-galactopyranoside	1.77	1.11–2.84	.02	1.76	1.06–2.92	0.03
D-threitol	2.74	1.04–7.20	.04	2.92	0.93–9.18	0.07
Tyrosine	0.59	0.35–0.98	.04	0.52	0.31–0.88	0.02
D-mannitol	1.28	0.84–1.98	0.09	1.26	0.99–1.60	0.06
Myo-inositol	2.92	0.85–1.33	.08	3.57	0.95–13.4	0.06

Models were adjusted for batch, sex,age, eGFR and DM.

* HR and SHR per 1 unit log base 2

For the ESRD events (Table 5), lactose, 2-O-glycerol-α-d-galactopyranoside, D-threitol and tyrosine were associated to the risk of ESRD, with tyrosine showing an inverse relationship. To account for the effect of competing risks, we performed Fine and Gray models for ESRD events and the subdistribution hazards are also shown in Table 5. With this approach, lactose, 2-O-glycerol-α-d-galactopyranoside, and tyrosine remained significantly associated to ESRD with similar estimates to those observed in the Cox models, while D-threitol, mannitol and myo-inositol presented borderline associations to this event.

## Discussion

In this study, we investigated the association between blood metabolites and the risk of overall death and ESRD in the Progredir Study, a cohort characterized by class 3 and 4 CKD, older patients and a high percentage of diabetics (57%). Our results disclosed a set of metabolites associated with the studied end-points.

D-malic acid, acetohydroxamic acid, butanoic acid, ribose, glutamine, trans-aconitic acid, lactose and an unidentified molecule (m/z 273) were all positively related to the risk of overall mortality, while docosahexaenoic acid, an omega-3 essential fatty acid, was inversely related to this risk, even after adjustments for age, sex, eGFR and diabetes.

D-malic acid can be found in fruits and herbs and is an intermediate in the butanoate metabolism, a pathway of energy metabolism used by colon bacteria [15], as is butanoic acid, a metabolite also related to the risk of death in our cohort. The fact that two metabolites of the same pathway were related to the outcome is of note, although pathway analysis did not show a significant association when multiple comparison was taken into account. Acetohydroxamic acid is known for being a synthetic drug with properties of antagonism to bacterial enzyme urease. However, in our cohort no participant was taking this drug. Unfortunately, very little is known about acetohydroxamic acid in the metabolome, although it has been reported in plants [16]. It is also of note that acetohydroxamic acid did not present any meaningful level of correlation with any of the other associated metabolites, suggesting that it may be acting through a completely independent pathway (S1 Fig). Ribose can be obtained from diet or produced by the pentose phosphate metabolism, a pathway related to diabetic complications [17–19]. Ribose has been reported to be associated with diabetic retinopathy in a study evaluating metabolomics [20]. Nonetheless, our results suggest that ribose is a marker of worse outcome independently of diabetes status. Also of note, ribose’s strongest relationship is with docosahexaenoic acid, suggesting that these two metabolites may be modulating outcome risk through the same pathway. Trans-aconitic acid is a tricarboxylic acid and has been reported in the urine of patients with organic acidurias [21,22]. Docosahexaenoic acid is an omega-3 essential fatty acid mostly found in fish oil and higher circulating levels have been related to lower inflammatory biomarkers and lower incidence of diabetes [23], overall mortality [24,25], CVD [26–28] and atrial fibrillation [29]. In dialysis patients, docosahexaenoic acid has been shown to be low and associated to increased risk of cardiovascular events [30] and higher levels of docosahexaenoic acid have been positively related to eGFR in the general population [31]. Supplementation of omega-3 fatty acid is still polemic [32] but has been shown to have some beneficial effects in IgA nephropathy [33–35], on eGFR decline after myocardial infarction [36], and on blood pressure in CKD patients [37].

This is the first study evaluating the association between metabolomics and mortality in a CKD population. Previous studies have evaluated the association between the metabolome and the risk of overall death in general population cohorts. One recent study [38] showed that nine metabolites (cotinine, mannose, glycocholate, pregnendiol disulfate, α-hydroxyisovalerate, N-acetylalanine, andro-steroid monosulfate 2, uridine, and γ-glutamyl-leucine) were independently associated with all-cause mortality. Citrate was the only low-molecular weight metabolite related to overall death in a study using NMR-platform [39]. In a study evaluating metabolites related to longevity defined as reaching age above 80 years, isocitrate, aconitate and malate, all metabolites related to the citric acid cycle, were inversely associated to longevity and positively related to all-cause and cardiovascular mortality [40]. Interestingly, in our findings malic acid and aconitic acid were positively related to the risk of death. However, the isomers associated in our data were D-malic acid and trans-aconitic acid, which are not the isomers occurring in the TCA cycle.

When only ESRD events were analyzed, lactose, 2-O-glycerol-α-d-galactopyranoside, and tyrosine were associated to its risk, while D-threitol, mannitol and myo-inositol presented borderline associations. With the exception of tyrosine, these metabolites share some properties: they can all be obtained from diet, are fermented by colonic microbioma [41, 42] and are related to energy metabolism. Interestingly, lactose, D-threitol and myo-inositol are all related to the galactose pathway. Also, they are strongly correlated in our Gaussian graphical model (S1 Fig).

Tyrosine is a semi-essential aminoacid that can either be obtained from diet or be derived from phenylalanine through the activity of phenylalanine hydroxylase, further used for synthesis of proteins, neurotransmitters and hormones, such as thyroid and adrenergic hormones. It has been shown that the kidney is the major source of tyrosine to the systemic circulation [43] and CKD is associated to decreasing levels of tyrosine in plasma and tissue, while phenylalanine and its toxic metabolites do not change or build-up [44]. Our data corroborates this finding since tyrosine was positively related to eGFR. Moreover, tyrosine was related to ESRD risk, raising interesting questions on the biological effects of decreasing tyrosine stores in CKD and the possible role of tyrosine supplementation in this population.

Our results are somewhat different from those previously reported by studies evaluating renal outcomes and metabolomics. This fact may be related to several factors. Differences in the metabolomics platform used and therefore on quantity and type of metabolites identified, as well as differences in analytical procedures and data processing, can all contribute to variability in the observed results. Race may also be a factor, since this is the only study so far performed in a Latin-American population, in contrast to results reported in European and North-American samples. In addition, most studies have addressed incident CKD, usually defined as a decline in eGFR or appearance of albuminuria between two time points. Only two studies evaluated ESRD or other hard outcome as end-points, and these were conducted in samples exclusively composed of diabetic patients [8,9]. Moreover, most studies were performed in populations free of CKD at baseline, with few evaluating participants with established CKD at study entry [7–9]. It is possible that the association between metabolites and the evaluated outcome changes dynamically as eGFR decreases, particularly considering that renal function presents such a major effect on metabolite concentration. This last argument also highlights our incomplete understanding of the mechanisms by which identified molecules mediate an increased risk of outcomes. However, it is tempting to suggest that the identified molecules point towards new pathways that may be target of pharmacological modulation in the CKD scenario.

Our study has some limitations. First, our sample size is moderate and possibly underpowered for smaller differences among groups. In addition, particularly for the ESRD analysis, we could not run more adjustments, considering the relatively small number of events. Second, many of the metabolites found to be associated to events, particularly those related to ESRD, showed a very significant association to baseline (creatinine-based) eGFR [14]. This implies that despite all models being adjusted for baseline eGFR, we cannot rule out the possibility that the relationship observed is still being at least partially determined by the fact that these metabolites may reflect renal function at baseline, and could, thus, be seen as a result due to reverse causation. Nonetheless, this is a fundamental challenge for all metabolomics studies in nephrology research. Whether some of the metabolites with top associations can be used as more accurate biomarkers of renal function is an interesting hypothesis under investigation [1, 14]. In addition, although we do show results for pathway analysis here, these findings are also limited by the main reasons mentioned above: our cohort is somewhat underpowered for capturing smaller effects and providing a large number of metabolites for pathway analysis, and the confounding effect of eGFR remains as a problem even after adjustments, considering the fact that all our participants present CKD. While exploring metabolite pathways and biochemical classes might provide interesting insights into the biology underlying the association of metabolites to kidney function, epidemiological studies in CKD populations may not be the best study design to assess that.

In conclusion, our data show metabolites related to the risk of death and ESRD in a CKD cohort. Although replication is needed, these findings raise interesting questions on the role of these metabolites as biomarkers and on the biological mechanisms underlying the observed relationships.

## Supporting information

S1 Table Full list of Cox regression models adjusted only for batch of the 265 metabolites identified on the risk of composite outcome (n = 129). (PDF)

S2 Table Pathway analysis for 34 selected metabolites significantly related to the composite outcome in the Progredir Cohort Study. (PDF)

S3 Table Correlation matrix for selected metabolites related to the composite outcome even after adjustments. (PDF)

S1 Fig Gaussian graphical model contemplating all composite outcome-associated metabolites, the composite outcome, and model covariates (sex, age, diabetes and CKDEPI eGFR) in the Progredir Cohort. (PDF)
