# Supplementary material for: Metabolomics biomarkers and the risk of overall mortality and ESRD in CKD: Results from the Progredir Cohort
Source: PLoS One. 2019 Mar 18;14(3):e0213764. doi: 10.1371/journal.pone.0213764 (PMC6422295; doi:10.1371/journal.pone.0213764)
Supplement: S1 Table — (PDF) [file pone.0213764.s001.pdf]

S1 Table. Full list of Cox regression models adjusted only for batch of the 265 metabolites identified on the risk of composite outcome (n=129).

	HMDB	KEGG	PubChem	HR*	p value	FDR q values
Lactose	HMDB0000186	C00243	84571	1.57	8.30E-12	**2.43E-09**
D-threitol	HMDB0004136	C16884	169019	2.46	9.09E-11	**1.33E-08**
Pseudouridine	HMDB0000767	C02067	15047	2.05	3.15E-09	**2.58E-07**
Butanoic acid	HMDB0000039	C00246	264	1.83	3.52E-09	**2.58E-07**
D-mannitol	HMDB0000765	C00392	6251	1.36	9.17E-08	**5.37E-06**
Trans-aconitic acid	HMDB0000958	C02341	444212	2.07	4.41E-07	**2.15E-05**
Acetohydroxamic acid	HMDB0014691	C06808	1990	2.06	1.56E-06	**6.53E-05**
Galactonic acid	HMDB0000565	C00880	128869	1.62	5.11E-06	**1.87E-04**
Myo-inositol	HMDB0000211	C00137	-	1.97	6.09E-06	**1.98E-04**
L-threonine	HMDB0000167	C00188	6288	0.60	7.78E-06	**2.28E-04**
2-O-Glycerol-α-D-galactopyranoside	-	-	-	1.54	3.78E-05	**1.01E-03**
Galacturonic acid	HMDB0002545	C08348	84740	1.57	5.34E-05	**1.30E-03**
L-glutamine	HMDB0000641	C00064	5961	1.65	6.58E-05	**1.39E-03**
Xylitol	HMDB0002917	C00379	6912	1.51	6.64E-05	**1.39E-03**
Gluconic acid	HMDB0000625	C00257	10690	1.79	1.29E-04	**2.52E-03**
5-hydroxyindol	HMDB0001855		9061	1.37	1.59E-04	**2.90E-03**
Unidentified m/z 405	-	-	-	1.56	1.71E-04	**2.95E-03**
Ribose	HMDB0000283	C00121	5779	1.40	2.33E-04	**3.80E-03**
p-Cresol glucuronide	HMDB0011686	-	154035	1.30	2.74E-04	**4.22E-03**
Tyrosine	HMDB0000158	C00082	6057	0.67	3.26E-04	**4.77E-03**
(S)-3,4-Dihydroxybutyric acid	HMDB0000337	-	150929	1.74	3.83E-04	**5.34E-03**

L-serine	HMDB0000187	C00065	5951	1.56	5.82E-04	**7.74E-03**
p-Hydroxyphenylacetic acid	HMDB0000020	C00642	127	1.28	9.19E-04	**1.17E-02**
Phenol	HMDB0000228	C00146	996	1.27	1.14E-03	**1.40E-02**
Eicosapentaenoic acid	HMDB0001999	C06428	446284	1.39	1.25E-03	**1.46E-02**
Unidentified m/z 273	-	-	-	1.93	1.96E-03	**2.21E-02**
Ribonic acid	HMDB0000867	C01685	5460677	1.51	2.21E-03	**2.40E-02**
D-malic acid	HMDB0031518	C00497	92824	1.52	2.54E-03	**2.66E-02**
Unidentified m/z 296	-	-	-	1.57	3.15E-03	**3.18E-02**
L-proline	HMDB0000162	C00148	145742	0.73	4.33E-03	**4.23E-02**
Acetamide	HMDB0031645	C06244	178	1.56	5.16E-03	**4.87E-02**
p-cresol	HMDB0001858	C01468	2879	1.34	5.32E-03	**4.87E-02**
Doconexent (docosahexaenoic acid)	HMDB0002183	C06429	445580	0.63	5.64E-03	**5.01E-02**
Threonic acid	HMDB0000943	C01620	151152	1.52	6.44E-03	**5.55E-02**
L-tryptophan 2	HMDB0000929	C00078	6305	0.73	7.39E-03	0.06
DL-isoleucine 2	-	-	791	0.76	7.93E-03	0.06
Carbonic acid, octadecyl vinyl ester	-	-	91693138	1.39	9.16E-03	0.07
(R*,S*)-2,3-Dihydroxybutanoic acid	HMDB0000498	-	13120901	1.34	1.07E-02	0.08
Benzoic acid, 4-ethoxy-, ethyl ester	HMDB0059898	-	90232	1.24	1.08E-02	0.08
Quinic acid	HMDB0003072	C06746	6508	1.23	1.11E-02	0.08
Phosphoric acid	HMDB0002142	C00009	1004	0.71	1.21E-02	0.09
Gluconic acid	HMDB0000625	C00257	10690	1.49	1.41E-02	0.10
Octadecane	HMDB0033721		11635	1.37	2.03E-02	0.14
Arabinose	HMDB0000646	C00259	439195	1.33	2.08E-02	0.14
D-Arabino-Hexonic acid	-	-	-	0.70	2.46E-02	0.16
Glycerol	HMDB0000131	C00116	753	0.77	2.68E-02	0.17
L-threonine 2	HMDB0000167	C00188	6288	0.74	2.76E-02	0.17
11-Methyldodecanol	-	-	33865	1.30	2.94E-02	0.17
(R*,R*)-2,3-Dihydroxybutanoic acid	HMDB0000498		13120901	1.23	2.95E-02	0.17
Creatinine	HMDB0000562	C00791	588	1.30	3.01E-02	0.17
Tetradecanoic acid	HMDB0000806	C06424	11005	0.59	3.14E-02	0.18

D-glucose 2	HMDB0000122	C00031	5793	15.76	3.49E-02	0.19
1,5-anhydro-D-sorbitol	HMDB0002712	C07326	64960	0.81	3.64E-02	0.19
1'-Oxocannabinol	-	-	6426272	0.88	3.65E-02	0.19
Galactitol	HMDB0000107	C01697	11850	1.17	3.70E-02	0.19
Elaidic acid	HMDB0000573	C00712	445639	1.24	3.74E-02	0.19
Unidentified m/z 167	-	-	-	0.83	4.87E-02	0.24
Succinic acid	HMDB0000254	C00042	1110	1.27	4.98E-02	0.24
L-glutamic acid 2	HMDB0000148	C00025	33032	0.81	5.09E-02	0.24
Dihydrouracil	HMDB0000076	C00429	649	1.44	5.67E-02	0.27
4-Bromo-1-butanol	-	-	118388	1.37	6.02E-02	0.28
L-cystine 3	HMDB0000192	C00491	67678	1.16	6.15E-02	0.28
Norvaline 2	HMDB0013716	C01799	439575	0.75	6.25E-02	0.28
L-leucine 2	HMDB0000687	C00123	6106	0.76	6.37E-02	0.28
Heneicosane	HMDB0061782	-	12403	1.33	6.69E-02	0.29
Glycine	HMDB0000123	C00037	750	0.82	6.81E-02	0.29
Ribitol	HMDB0000508	C00474	-	1.24	7.27E-02	0.30
Undecane	HMDB0031445	-	14257	1.26	7.29E-02	0.30
2-Propenoic acid	HMDB0031647	C00511	6581	1.59	7.41E-02	0.30
Anthraquinone, 1-o-chlorophenyl	-	-	624935	0.87	8.04E-02	0.32
Pyroglutamic acid	HMDB0000267	C01879	7405	1.32	9.38E-02	0.37
Decane	HMDB0031450	-	15600	1.23	1.05E-01	0.40
L-Phenylalanine	HMDB0000159	C00079	6140	0.79	1.06E-01	0.40
Propylene glycol	HMDB0001881	C00583	1030	1.10	1.23E-01	0.45
Unidentified m/z 302	-	-	-	1.19	1.27E-01	0.47
Eicosane	HMDB0059909	-	8222	1.39	1.30E-01	0.47
5alpha-Cholestanol	HMDB0000908	-	6665	1.28	1.33E-01	0.47
Ethanol, 2-methylamino, N-trifluoroacetyl	-	-	57161818	1.95	1.34E-01	0.47
L-alanine 1	HMDB0000161	C00041	5950	0.86	1.35E-01	0.47
L-serine 2	HMDB0000187	C00065	5951	0.80	1.36E-01	0.47

Glycolic acid	HMDB0000115	C00160	757	1.20	1.37E-01	0.47
2-ketoisocaproic acid 2	HMDB0000695	C00233	70	0.89	1.40E-01	0.47
D-glucose 1	HMDB0000122	C00031	5793	0.74	1.41E-01	0.47
L-norleucine 1	HMDB0001645	C01933	21236	0.82	1.43E-01	0.47
Diethanolamine	HMDB0004437	C06772	8113	1.15	1.48E-01	0.48
Methyl galactoside	HMDB0029965	-	2108	1.59	1.49E-01	0.48
1-Nonene	HMDB0031270	C08452	31285	1.14	1.49E-01	0.48
N-Hydroxymethyl-trifluoroacetamide	-	-	3084931	1.37	1.57E-01	0.49
Palmitoleic acid	HMDB0003229	C08362	445638	0.90	1.70E-01	0.53
3-Pyridinol	-	-	7971	1.19	1.72E-01	0.53
Glycine	HMDB0000123	C00037	750	1.14	1.73E-01	0.53
L-cystine 1	HMDB0000192	C00491	67678	1.15	1.78E-01	0.53
L-methionine 2	HMDB0000696	C00073	6137	0.84	1.80E-01	0.53
Unidentified m/z 526	-	-	-	1.15	1.86E-01	0.54
L-Alanine	HMDB0000161	C00041	5950	0.81	1.88E-01	0.54
Tetradecane, 1-iodo	-	-	29507	1.36	1.89E-01	0.54
N-acetyl-D-glucosamine 2	HMDB0000215	C00140	439174	1.26	1.92E-01	0.54
Hexadecane	HMDB0033792	C14499	11006	1.51	1.92E-01	0.54
L-Valine	HMDB0000883	C00183	6287	0.81	2.02E-01	0.56
10-Heptadecenoic acid, (Z)-	HMDB0060038	-	5312435	0.81	2.03E-01	0.56
Undecane, 2,10-dimethyl-	-	-	519386	1.26	2.16E-01	0.58
Thiazolidine-2,5-dione	-	-	542718	1.11	2.16E-01	0.58
2-(Methylamino)ethanol	-	-	8016	1.21	2.19E-01	0.58
1 monoolein	-	-	-	1.22	2.23E-01	0.59
beta.-D-Galactofuranoside	-	-	-	1.24	2.24E-01	0.59
Dodecane, 4,6-dimethyl	-	-	-	1.19	2.29E-01	0.59
Oxalic acid	HMDB0002329	C00209	971	1.07	2.30E-01	0.59
DL-isoleucine 1	-	-	791	0.78	2.35E-01	0.60
L-Proline	HMDB0000162	C00148	145742	1.35	2.38E-01	0.60
Citric acid	HMDB0000094	C00158	311	1.23	2.42E-01	0.60

Glycolic acid	HMDB0000115	C03547	757	1.23	2.44E-01	0.60
1-stearoyl-rac-glycerol	HMDB0031075		24699	1.85	2.45E-01	0.60
2-Pyrrolidinone	HMDB0002039	C11118	12025	1.24	2.46E-01	0.60
1-Hexadecanol	HMDB0003424	C00823	2682	1.11	2.48E-01	0.60
Benzoic acid	HMDB0001870	C00180	243	0.81	2.51E-01	0.60
Dodecane, 1-iodo	-	-	20282	1.18	2.54E-01	0.60
N-(6-Quinolinyl)phthalimide	-	-	623675	1.14	2.59E-01	0.60
Citrulline 2	HMDB0000904	C00327	9750	1.14	2.62E-01	0.60
Psicose 1		-	441036	1.10	2.62E-01	0.60
3-indolelactic acid 2	HMDB0000671	C02043	92904	1.15	2.62E-01	0.60
Xylose 1	HMDB0000098	C00181	135191	1.24	2.65E-01	0.60
D-(+)-Turanose	HMDB0011740	C19636	5460935	1.37	2.66E-01	0.60
DL-Ornithine	HMDB32455	C01602	389	0.88	2.70E-01	0.60
Heptacosane	-	-	11636	1.20	2.73E-01	0.60
Linoleic acid	HMDB0000673	C01595	5280450	1.19	2.73E-01	0.60
2-hydroxybutyric acid	HMDB0000008	C05984	11266	1.22	2.74E-01	0.60
Uric acid 1	HMDB0000289	C00366	1175	1.66	2.78E-01	0.60
1-Decanol	HMDB0011624	C01633	8174	1.12	2.80E-01	0.60
Undecane, 3,8-dimethyl	-	-	-	1.14	2.92E-01	0.62
2-.alpha.-Mannobiose	-	-	91746263	1.18	2.95E-01	0.62
Unidentified m/z 338	-	-	-	1.99	2.96E-01	0.62
Tagatose 1	HMDB0003418	C00795	92092	1.13	2.99E-01	0.62
1H-Pyrazole, 4-nitro	-	-	-	1.19	2.99E-01	0.62
2-Palmitoylglycerol	-	-	123409	0.85	2.99E-01	0.62
Guanosine 2	HMDB0000133	C00387	6802	0.89	3.09E-01	0.63
Tetramethylbenzene	HMDB0059823	-	10263	0.86	3.09E-01	0.63
Ethanolamine	HMDB0000149	C00189	700	0.89	3.14E-01	0.63
trans-4-hydroxy-L-proline 1	HMDB0000725	C01157	5810	1.09	3.14E-01	0.63
9-Hexadecenoic acid, (Z)	HMDB0003229	C08362	445638	0.87	3.25E-01	0.64
2-Phenyl-1,3-oxazol-2-ine	-	-	249990821	0.93	3.26E-01	0.64

Tetracosane	-	-	12592	1.20	3.27E-01	0.64
beta-Gentiobiose	-	-	441422	1.09	3.27E-01	0.64
L-methionine 1	HMDB0000696	C00073	6137	0.90	3.44E-01	0.66
Unidentified m/z 381	-	-	-	1.07	3.45E-01	0.66
Phosphoric acid 2	HMDB0002142	C00009	1004	0.89	3.45E-01	0.66
3-alpha-Mannobiose	-	-	-	0.87	3.52E-01	0.66
1-Dodecanol	HMDB0011626	C02277	8193	1.17	3.56E-01	0.66
L-(+) lactic acid	HMDB0000190	C00256	61503	0.90	3.56E-01	0.66
Pyrophosphate	HMDB0000250	C00013	644102	0.94	3.57E-01	0.66
2-Methoxy-6-methyl-4-phenyl-quinazoline	-	-	760760	1.18	3.58E-01	0.66
Glycine	HMDB0000123	C00037	750	1.12	3.66E-01	0.66
O-phosphocolamine	HMDB0000224	C00346	1015	1.14	3.67E-01	0.66
Methoxyamine	HMDB0014861	C07513	6082	0.89	3.69E-01	0.66
Hypoxanthine	HMDB0000157	C00262	790	1.10	3.81E-01	0.68
2-Hydroxybutyric acid	HMDB0000008	C05984	11266	0.92	3.84E-01	0.68
Unidentified m/z 536	-	-	-	1.06	3.89E-01	0.69
Propylparaben	HMDB0032574		7175	0.88	4.04E-01	0.70
dibenzo-1,4,8,11-tetraazacyclotetradecine, 5,6,7,8,9,14,15,16,17,18-decahydro	-	-	-	1.16	4.08E-01	0.71
(t-Butyldimethylsilyl)[3-methyl-3-(4-methyl-pent-3-enyl)-oxiran-2-yl]-methanone	-	-	-	1.13	4.28E-01	0.73
Dioctyl phthalate	-	-	8346	1.12	4.29E-01	0.73
2-Monostearin	-	-	79075	0.89	4.33E-01	0.73
m-Cresol	HMDB0002048	C01467	342	0.92	4.35E-01	0.73
Stearic acid	HMDB0000827	C01530	5281	1.26	4.45E-01	0.74
1-hexadecanol	HMDB0003424	C00823	2682	0.87	4.46E-01	0.74
beta-Tocopherol	HMDB0006335	C14152	6857447	1.09	4.82E-01	0.79
Butylphosphonic acid	-	-	76839	1.06	4.87E-01	0.80

1-(Trifluoroacetyl)-L-proline	-	-	11082665	1.13	4.93E-01	0.80
Glycerol 1-phosphate	HMDB0000126	C00093	439162	1.08	4.96E-01	0.80
Methyl linolenate	HMDB0034381	-	5319706	1.17	4.96E-01	0.80
Heptadecanoic acid, glycerine-(1)-monoester	-	-	633500	0.87	5.01E-01	0.80
1-Heptanol	HMDB0031479		8129	1.12	5.14E-01	0.81
Aminomalonic acid	HMDB0001147	C00872	100714	0.93	5.21E-01	0.82
Arachidic acid	HMDB0002212	C06425	10467	1.09	5.21E-01	0.82
L-lysine 1	HMDB0000182	C00047	5962	1.13	5.25E-01	0.82
Xanthine	HMDB0000292	C00385	1188	1.05	5.30E-01	0.82
1-Hydroxy-3-methoxy-6-methylanthraquinone	-	-	179389	1.06	5.31E-01	0.82
L-asparagine 2	HMDB0000168	C00152	6267	0.93	5.36E-01	0.82
L-glutamic acid 3	HMDB0000148	C00025	33032	0.61	5.57E-01	0.84
Acetyl chloride	-	-	6367	0.92	5.65E-01	0.84
Phosphoric acid	HMDB0002142	C00009	1004	0.95	5.65E-01	0.84
Glucose	HMDB0000122	C00031	5793	1.04	5.67E-01	0.84
Lauric acid	HMDB0000638	C02679	3893	1.11	5.76E-01	0.84
alpha-D-Glucopyranose	HMDB0003345	C00267	79025	0.96	5.81E-01	0.84
Unidentified m/z 334	-	-	-	1.14	5.86E-01	0.84
L-tyrosine 2	HMDB0000158	C00082	6057	1.06	5.87E-01	0.84
1,1'-Biphenyl, 4,4'-dinitro	-	-	-	1.15	5.88E-01	0.84
3-Bromo-1-propanol	-	-	12308	1.07	5.94E-01	0.84
1-Octadecanol	HMDB0002350	D01924	8221	1.11	5.96E-01	0.84
L-cystine 2	HMDB0000192	C00491	67678	1.06	5.96E-01	0.84
Beta- alanine 1	HMDB0000056	C00099	239	0.91	5.97E-01	0.84
2,6-Diisopropylnaphthalene	-	-	32241	1.05	6.03E-01	0.84
L-valine 2	HMDB0000883	C00183	6287	0.97	6.05E-01	0.84
L-Alanine	HMDB0000161	C00041	5950	0.94	6.19E-01	0.86
1-Dodecanol	HMDB0011626	C02277	8193	1.07	6.23E-01	0.86

N-Acetyl glucosamine methoxime	-	-	9601827	0.97	6.26E-01	0.86
9-Tetradecenoic acid, (E)	HMDB0062248	-	5312402	0.94	6.28E-01	0.86
beta-D-Galactofuranose	-	-	11019448	1.09	6.32E-01	0.86
5,5'-Biphthalide	-	-	617084	0.96	6.36E-01	0.86
Arachidonic acid	HMDB0001043	C00219	444899	0.95	6.38E-01	0.86
L-Proline	HMDB0000162	C00148	145742	1.06	6.44E-01	0.86
L-lysine 2	HMDB0000182	C00047	5962	0.97	6.45E-01	0.86
Unidentified m/z 415	-	-	-	0.93	6.48E-01	0.86
1-Monomyristin	-	-	249965408	0.92	6.55E-01	0.86
13-Docosenoic acid, methyl ester, (Z)	-	-	5364423	1.12	6.60E-01	0.87
Pentadecanoic acid	HMDB0000826	C16537	13849	0.94	6.68E-01	0.87
Uric acid 2	HMDB0000289	C00366	1175	1.07	6.69E-01	0.87
Hexadecane	HMDB0033792	C14499	11006	0.96	6.77E-01	0.87
Galactose oxime	-	-	135055079	1.04	6.88E-01	0.88
1-octadecene	-	-	8217	0.96	6.96E-01	0.88
1,2,5-Thiadiazolo[3,4-c]coumarine, 8-nitro	-	-	250100269	1.10	6.98E-01	0.88
2,4-Thiazolidinedione	-	-	5437	1.03	7.00E-01	0.88
Tricosanoic acid	HMDB0001160	-	17085	0.97	7.11E-01	0.89
Phenylalanine 1	HMDB0000159	C00079	6140	1.05	7.15E-01	0.89
L-glutamine 1	HMDB0000641	C00064	5961	1.05	7.18E-01	0.89
9-Decenoic acid	HMDB0031003	-	61743	0.93	7.24E-01	0.90
Tyrosine 2	HMDB0000158	C00082	6057	1.03	7.34E-01	0.90
1-Monopalmitin	HMDB0011564	-	3084463	1.05	7.38E-01	0.90
Cholesterol	HMDB0000067	C00187	11025495	0.95	7.54E-01	0.92
D-allose 1	HMDB0001151	C01487	12285879	1.03	7.66E-01	0.93
Palmitic acid	HMDB0000220	C00249	985	1.11	7.68E-01	0.93
Urea	HMDB0000294	C00086	1176	0.96	7.74E-01	0.93
L-glutamic acid 1	HMDB0000148	C00025	33032	1.04	7.78E-01	0.93
Heptadecanoic acid	HMDB0002259		10465	0.97	7.81E-01	0.93

Capric acid	HMDB0000511	C01571	2969	1.05	7.81E-01	0.93
Cholest-7-en-3-ol, (3.beta.,5.alpha.)	-	-	-	1.04	7.91E-01	0.93
Oxalic acid	HMDB0002329	C00209	971	1.02	7.93E-01	0.93
Nonanoic acid	HMDB0000847	C01601	8158	0.96	8.05E-01	0.94
Ethyl .alpha.-D-glucopyranoside	-	-	9815668	0.97	8.17E-01	0.94
1,2-Benzenedicarboxylic acid	HMDB0002107	C01606	1017	1.04	8.18E-01	0.94
Propanoic acid	HMDB0000237	C00163	1032	0.99	8.22E-01	0.94
2-Monomyristin	HMDB0011530	-	137938	0.96	8.25E-01	0.94
Inosine	HMDB0000195	C00294	6021	0.98	8.32E-01	0.94
Methyl 2-[2-(4-chlorophenyl)-5-methyl-1H-imidazol-1-yl]dithiobenzoate	-	-	628399	0.98	8.32E-01	0.94
2-Methoxyestradiol	HMDB0000405	C05302	66414	0.98	8.35E-01	0.94
Phenol	HMDB0000228	C15584	996	1.03	8.44E-01	0.95
Carbonic acid	HMDB0003538	C01353	767	1.02	8.51E-01	0.95
2,3-Dihydroxypropyl icosanoate	-	-	-	1.03	8.51E-01	0.95
4-Pyridinol	-	-	12290	1.03	8.53E-01	0.95
Benzoic Acid	HMDB0001870	C00180	243	1.03	8.53E-01	0.95
Uridine	HMDB0000296	C00299	6029	0.97	8.55E-01	0.95
gamma-Tocopherol	HMDB0001492		14986	1.02	8.59E-01	0.95
L-Asparagine	HMDB0000168	C00152	6267	0.97	8.62E-01	0.95
3-.alpha.-Mannobiose	-	-	-	0.98	8.69E-01	0.95
Monolaurin	-	-	14871	0.97	8.80E-01	0.95
Aspartic acid 2	HMDB0000191	C00049	5960	0.98	8.85E-01	0.95
Uridine	HMDB0000296	C00299	6029	0.97	8.85E-01	0.95
Pentadecanoic acid, glycerine-(1)-monoester	-	-	-	1.03	8.85E-01	0.95
L-alanine 2	HMDB0000161	C00041	5950	0.97	8.86E-01	0.95
Phosphoric acid	HMDB0002142	C00009	1004	1.01	8.95E-01	0.95
3-indoleacetic acid	HMDB0000197	C00954	802	0.99	9.09E-01	0.97

1,2-Dipalmitin	-	-	99931	1.01	9.17E-01	0.97
2-Ethyl-3-hydroxypropionic acid	HMDB0000396	-	188979	1.01	9.19E-01	0.97
L-Serine	HMDB0000187	C00065	5951	1.01	9.24E-01	0.97
Ethanolamine	HMDB0000149	C00189	700	0.99	9.27E-01	0.97
2-Hydrazino-4,6-dimethylpyrimidine	-	-	-	1.01	9.29E-01	0.97
Aspartic acid 1	HMDB0000191	C00049	5960	0.99	9.51E-01	0.98
Lactic Acid	HMDB0000190	C00186	107689	1.00	9.58E-01	0.99
Glyceric acid	HMDB0000139	C00258	439194	0.99	9.59E-01	0.99
Methyl palmitoleate	-	-	643801	1.01	9.70E-01	0.99
alpha-Tocopherol	HMDB0001893	C02477	14985	0.99	9.73E-01	0.99
Serotonin 1	HMDB0000259	C00780	5202	1.00	9.77E-01	0.99
Sedoheptulose	HMDB0003219	C02076	102926	1.00	9.80E-01	0.99
2,6-Bis(tert-butyl)phenol	-	-	31405	1.00	9.84E-01	0.99
1,3-Dipalmitin	HMDB0031011	-	68149	1.00	9.90E-01	0.99
Leucyltryptophan	HMDB0028940	-	329275	1.00	9.92E-01	0.99

Models were adjusted for batch

* HR per 1 unit log base 2
