# Supplementary material for: Metabolomics biomarkers and the risk of overall mortality and ESRD in CKD: Results from the Progredir Cohort
Source: PLoS One. 2019 Mar 18;14(3):e0213764. doi: 10.1371/journal.pone.0213764 (PMC6422295; doi:10.1371/journal.pone.0213764)
Supplement: S2 Table — (PDF) [file pone.0213764.s002.pdf]

S2 Table. Pathway analysis for 34 selected metabolites significantly related to the composite outcome in the Progredir Cohort Study.

	Total	Hits	Raw p	FDR	Impact
Aminoacyl-tRNA biosynthesis	75	5	0.001	0.10	0.06
Galactose metabolism	41	3	0.01	0.40	0.08
Pentose phosphate pathway	32	2	0.05	0.89	0.09
Tyrosine metabolism	76	3	0.05	0.89	0.11
Nitrogen metabolism	39	2	0.07	0.89	0.00
Butanoate metabolism	40	2	0.07	0.89	0.03
Phenylalanine metabolism	45	2	0.09	0.89	0.00
Ascorbate and aldarate metabolism	45	2	0.09	0.89	0.01
Glycine, serine and threonine metabolism	48	2	0.10	0.89	0.23
D-Glutamine and D-glutamate metabolism	11	1	0.12	0.94	0.03
Pyrimidine metabolism	60	2	0.14	1.00	0.00
Cyanoamino acid metabolism	16	1	0.17	1.00	0.00
Sulfur metabolism	18	1	0.18	1.00	0.00
Arginine and proline metabolism	77	2	0.21	1.00	0.10
Thiamine metabolism	24	1	0.24	1.00	0.00
Alanine, aspartate and glutamate metabolism	24	1	0.24	1.00	0.21
Sphingolipid metabolism	25	1	0.25	1.00	0.00
Valine, leucine and isoleucine biosynthesis	27	1	0.26	1.00	0.00
Phenylalanine, tyrosine and tryptophan biosynthesis	27	1	0.26	1.00	0.01
Methane metabolism	34	1	0.32	1.00	0.02
Ubiquinone and other terpenoid-quinone biosynthesis	36	1	0.34	1.00	0.00
Inositol phosphate metabolism	39	1	0.36	1.00	0.14
Fructose and mannose metabolism	48	1	0.42	1.00	0.00
Pentose and glucuronate interconversions	53	1	0.45	1.00	0.03
Cysteine and methionine metabolism	56	1	0.47	1.00	0.01
Purine metabolism	92	1	0.65	1.00	0.00
Porphyrin and chlorophyll metabolism	104	1	0.70	1.00	0.00
