# Supplementary material for: Metabolomics biomarkers and the risk of overall mortality and ESRD in CKD: Results from the Progredir Cohort
Source: PLoS One. 2019 Mar 18;14(3):e0213764. doi: 10.1371/journal.pone.0213764 (PMC6422295; doi:10.1371/journal.pone.0213764)
Supplement: S3 Table — (PDF) [file pone.0213764.s003.pdf]

S3 Table. Correlation matrix for selected metabolites related to the composite outcome even after adjustments.

	Lactose	Acetohydroxamic acid	D-threitol	Doconexent	Butanoic acid	D-mannitol	Trans-aconitic acid	Pseudo uridine	L-glutamine	L-threonine	Eicosapentaenoic acid	Ribose	D-malic acid	Unindentified	L-serine	p-Cresol glucuronide	galacturonic acid	2-O-Glycerol- α-d-galactopyranoside
Lactose	1.000	0.344	0.665	0.187	0.605	0.517	0.575	0.649	0.397	0.052	0.404	0.340	0.328	0.297	0.233	0.368	0.350	0.493
Acetohydroxamic acid	0.344	1.000	0.652	0.417	0.534	0.181	0.550	0.632	0.351	0.365	0.371	0.417	0.487	0.358	0.263	0.206	0.241	0.498
D-threitol	0.665	0.652	1.000	0.361	0.857	0.555	0.774	0.912	0.499	0.275	0.433	0.610	0.506	0.485	0.340	0.310	0.543	0.702
Doconexent	0.187	0.417	0.361	1.000	0.310	-0.068	0.336	0.415	0.094	0.282	0.185	0.101	0.352	0.159	0.023	-0.023	0.001	0.329
Butanoic acid	0.605	0.534	0.857	0.310	1.000	0.437	0.636	0.857	0.526	0.292	0.403	0.537	0.476	0.403	0.340	0.235	0.537	0.662
D-mannitol	0.517	0.181	0.555	-0.068	0.437	1.000	0.308	0.403	0.111	-0.202	0.201	0.206	0.095	0.211	0.133	0.296	0.293	0.243
Trans-aconitic acid	0.575	0.550	0.774	0.336	0.636	0.308	1.000	0.761	0.470	0.262	0.333	0.556	0.514	0.495	0.255	0.254	0.380	0.558
Pseudo uridine	0.649	0.632	0.912	0.415	0.857	0.403	0.761	1.000	0.559	0.273	0.460	0.512	0.502	0.577	0.359	0.323	0.558	0.706
L-glutamine	0.397	0.351	0.499	0.094	0.526	0.111	0.470	0.559	1.000	0.189	0.236	0.537	0.335	0.381	0.445	0.182	0.361	0.369
L-threonine	0.052	0.365	0.275	0.282	0.292	-0.202	0.262	0.273	0.189	1.000	0.195	0.357	0.394	0.100	0.318	-0.044	0.038	0.189
Eicosapentaenoic acid	0.404	0.371	0.433	0.185	0.403	0.201	0.333	0.460	0.236	0.195	1.000	0.065	0.279	0.300	0.194	0.082	0.134	0.347
Ribose	0.340	0.417	0.610	0.101	0.537	0.206	0.556	0.512	0.537	0.357	0.065	1.000	0.365	0.262	0.271	0.120	0.447	0.458
D-malic acid	0.328	0.487	0.506	0.352	0.476	0.095	0.514	0.502	0.335	0.394	0.279	0.365	1.000	0.353	0.325	0.215	0.093	0.307
Unindentified	0.297	0.358	0.485	0.159	0.403	0.211	0.495	0.577	0.381	0.100	0.300	0.262	0.353	1.000	0.217	0.164	0.206	0.329
L-serine	0.233	0.263	0.340	0.023	0.340	0.133	0.255	0.359	0.445	0.318	0.194	0.271	0.325	0.217	1.000	0.143	0.290	0.243
p-Cresol glucuronide	0.368	0.206	0.310	-0.023	0.235	0.296	0.254	0.323	0.182	-0.044	0.082	0.120	0.215	0.164	0.143	1.000	0.262	0.194
galacturonic acid	0.350	0.241	0.543	0.001	0.537	0.293	0.380	0.558	0.361	0.038	0.134	0.447	0.093	0.206	0.290	0.262	1.000	0.334
2-O-Glycerol-α-d-galactopyranoside	0.493	0.498	0.702	0.329	0.662	0.243	0.558	0.706	0.369	0.189	0.347	0.458	0.307	0.329	0.243	0.194	0.334	1.000
