# Supplementary material for: Metabolomics biomarkers and the risk of overall mortality and ESRD in CKD: Results from the Progredir Cohort
Source: PLoS One. 2019 Mar 18;14(3):e0213764. doi: 10.1371/journal.pone.0213764 (PMC6422295; doi:10.1371/journal.pone.0213764)
Supplement: S1 Fig — (PDF) [file pone.0213764.s004.pdf]

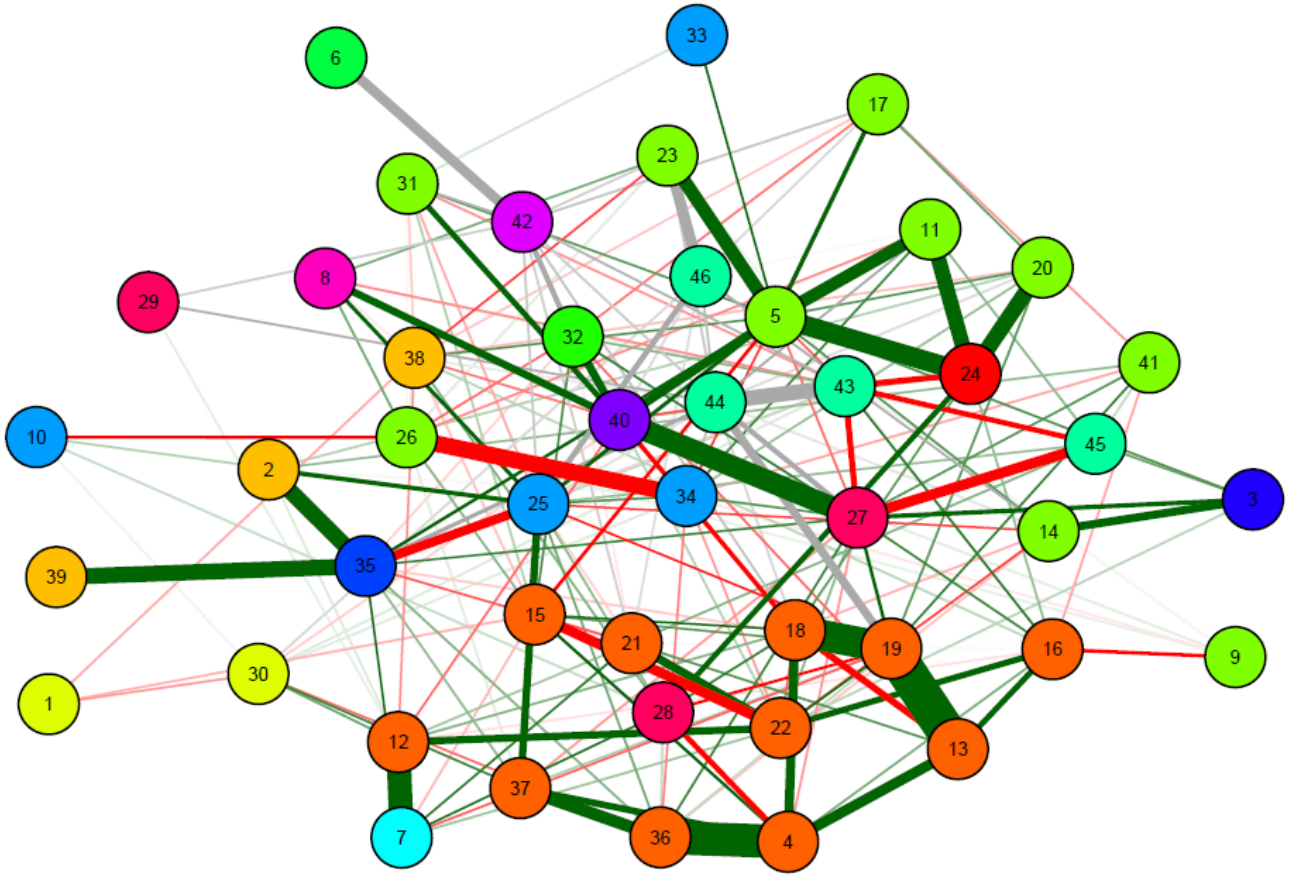

Alcohols and polyols
- 24: Myo−inositol

Amino acids
- 4: L−proline
- 12: L−serine 1
- 13: L−serine 2
- 15: Tyrosine 1
- 16: Tyrosine 2
- 18: L−threonine 1
- 19: L−threonine 2
- 21: L−glutamine 1
- 22: L−glutamine 2
- 36: L−proline 1
- 37: L−proline 2

Benzenoids
- 2: p−Hydroxyphenylacetic acid
- 38: Phenol 1
- 39: Phenol 2

Beta hydroxy acids
- 1: (S)−3,4−Dihydroxybutyric acid 1
- 30: (S)−3,4−Dihydroxybutyric acid 2

Carbohydrates
- 5: D−threitol
- 9: Galacturonic acid
- 11: Threonic acid
- 14: Gluconic acid
- 17: D−mannitol
- 20: Xylitol
- 23: Lactose
- 26: Ribose
- 31: 2−O−Glycerol−a−D−galactopyranoside
- 41: Ribonic acid

Carboximidic acids
- 32: Acetamide

Carboxylic acid
- 6: Acetohydroxamic acid

Covariate
- 43: CKD Epi
- 44: Sex
- 45: Age
- 46: Diabetes

Cresols
- 7: p−cresol

Fatty acids
- 10: Eicosapentaenoic acid
- 25: D−malic acid
- 33: Butanoic acid
- 34: Doconexent (docosahexaenoic acid)

Hydroxyindoles
- 35: 5−hydroxyindol

Medium−chain hydroxy acids
- 3: Galactonic acid

Nucleoside
- 40: Pseudouridine

Outcome
- 42: Combined Events

Tricarboxylic acids
- 8: Trans−aconitic acid

Unidentified
- 27: Unidentified m/z 405
- 28: Unidentified m/z 296
- 29: Unidentified m/z 273

S1 Fig. Gaussian graphical model contemplating all composite outcome-associated metabolites, the composite outcome, and model covariates (sex, age, diabetes and CKDEPI eGFR) in the Progredir Cohort. Green edges indicate positive weights and red edges indicate negative weights. Node colors follow biochemical class. The width of the edges corresponds to the absolute weight and scale relative to the strongest weight in the graph.
